# LC-MS Based Metabolomics Analysis of Potato (*Solanum tuberosum* L.) Cultivars Irrigated with Quicklime Treated Acid Mine Drainage Water

**DOI:** 10.3390/metabo12030221

**Published:** 2022-03-02

**Authors:** Rabelani Munyai, Maropeng Vellry Raletsena, David Mxolisi Modise

**Affiliations:** 1 Department of Agriculture and Animal Health, Florida Science Campus, University of South Africa, Roodepoort 1709, South Africa; raletmv@unisa.ac.za; 2 Faculty of Natural and Agricultural Sciences, Potchefstroom Campus, North West University, Private Bag X6001, Potchefstroom 2520, South Africa; dmmxo9@gmail.com

**Keywords:** acid mine drainage, irrigation, metabolites, potato, quicklime

## Abstract

In water-scarce areas, the reuse of (un)treated acid mine drainage (AMD) water for crop irrigation has become a requirement, but it also carries a wide range of contaminants that can elicit the synthesis of diverse metabolites necessary for the survival of the plants. There is still a paucity of studies on the impact of quicklime treated-AMD water on the metabolite synthesis of potatoes. This study examined the effect of the irrigation of two potato cultivars (Marykies and Royal cultivars) with quicklime-treated AMD water on their metabolite profiles. A greenhouse study was conducted with five experimental treatments with different solution ratios, replicated three times in a completely randomized design. A total of 40 and 36 metabolites from Marykies and Royal cultivars which include amino acids, organic acids, and aromatic amines were identified, respectively. The results revealed elevation in the abundance of metabolites under the irrigation with treated AMD water for both cultivars with subtle variations. This will provide information on the primary metabolite shifst in potato that enhance their survival and growth under AMD conditions. However, more specific data on toxicity due to AMD irrigation would be required for a refined risk assessment.

## 1. Introduction

Freshwater supply is one of the world’s biggest issues, with approximately one-third of the world’s drinking water coming from surface sources such as rivers, dams, lakes, and canals. South Africa, like many other countries, pollution of existing water resources is the most danger to a sustainable water supply [1]. The country is listed as one of the driest countries in the world, and serious water scarcity is projected to occur very soon [2]. The high population growth rate, industrialization, and urbanization have resulted in a global shortage of high-quality water supplies [3,4,5,6]. Rural and urban populations rely on surface water, and with the country’s economy heavily reliant on mining, the water supplies are progressively being polluted, notably by acid mine drainage (AMD) [5].

Acid mine drainage is one the major challenge affecting the availability of water both for domestic and agricultural uses. It is characterized by low levels of pH and high heavy metal concentrations [7,8]. AMD has excessive amounts of total dissolved solids, which represent an extreme environment which is unbearable to most life forms [9,10]. Furthermore, AMD is characterised by diverse microorganisms belonging to the following domains: *Bacteria*, *Archaea* and *Eukarya* [7,10,11,12]. Unfortunately, there is plenty of AMD water that is disposed into the environment without being treated for re-use [5]. Discharged AMD negatively affects existing water courses, soil fertility and crop output, as well as water delivery systems for drinking and irrigation, along with various infrastructures [5,13,14,15]. Heavy metal exposure at hazardous levels, due to AMD water use in agriculture, causes a wide range of physiological and metabolic changes in plants [16]. In high concentrations, heavy metals reduce plant growth, biomass output, protein content, and chlorophyll pigment synthesis, and potentially resulting in significant crop yield reductions [17]. For instance, in high concentrations, Cd, Pb and Cr have been reported to affect several metabolic processes in plants [18]. Even though mine water re-use could be one possible source of agricultural water, helping to save water resources and reduce environmental issues associated with effluent discharge into bodies of water, there are potential detrimental effects on crop production. Various studies have been performed on the potential of using mine water for irrigation, with varying levels of success [19,20,21,22,23,24]. However, irrigation with AMD water has been reported to have a direct impact on physiological performance in terms of the growth, metabolism, and reproduction of plants [25,26].

In recent years, the metabolomics phenomenon has been widely used to categorize how different cultivation methods, environments and irrigation types affect plants, as well as to assess the quality of agricultural products [27,28]. Metabolomics can detect a wide range of metabolites from a single extract, allowing for quick and precise metabolite analysis [29] and some of these metabolites have been linked to heavy metal stress tolerance levels. In addition, since metabolites have such a wide range of chemical properties, new functional other genomics technologies have evolved in recent years, including high-throughput transcriptomic, proteomic, metabolomic, and ionomic studies used to reveal plant biochemical responses [30,31,32]. Feng et al. 2020 [33] recently confirmed that the analysis of metabolites using plant metabolomic technologies can certainly provide information on how crop species respond to abiotic stress. A mass spectrometry (MS)-based metabolomic methodology has been integrated with a variety of analytical separation techniques, including gas chromatography (GC), liquid chromatography (LC), and capillary electrophoresis (CE), since it produces very sensitive results and allows for high-throughput data gathering [34]. Among other analytical separation techniques, LC-MS was used due to its unique properties that allow for direct probing of metabolites in any sample without the necessity of derivatization [35]. Several studies on plant metabolomics have been performed on various crops such as Zucchini [36], soybean [37], lettuce [38], and rice [39]. Plants can synthesize around 100,000 primary and secondary metabolites, of which only about 10% have been identified to date [40,41,42]. Metabolites have a variety of functions, including growth and development, respiration and photosynthesis, hormones, and protein synthesis, and are associated with increasing crop survival in stressful situations [42,43]. Primary metabolites in plants, such as amino acids, enzymes, and carbohydrates, keep plant growth processes running optimally and help plants to grow and develop [44,45]. Plants respond to stress by changing gene expressions, protein abundance, and through metabolite accumulation at the molecular level as defense mechanisms [33].

Potato (*S. tuberosum*) is a highly sensitive crop, in which an adequate supply of water is essential to achieve a high-quality yield [46]. However, there is limited or no work on the effect of the use of quicklime treated AMD irrigation on the metabolomics profiles of the Marykies and Royal cultivars. Since the pioneering study [47] on simultaneous analysis of metabolites in potato tubers by gas chromatography-mass spectrometry, there have been several publications on the potato tuber metabolome [48]. The current study thus aimed to compare the metabolic changes, using the LC-MS analytical technique, in Marykies and Royal potato cultivars when subjected to quicklime-treated AMD irrigation. This will provide the baseline information on the primary metabolites shifts in potatoes that enhance their survival and growth under AMD conditions.

## 2. Results

### 2.1. Metabolome Profile Variations in Two Cultivars of Potato under Irrigation with Treated AMD Water

Two potato cultivars (Marykies and Royal) were used for analysis in this study. Each cultivar had 45 samples from five treatments analyzed (Treatment 1(T1) = 0:0, tap water; Treatment 2(T2) = 0:100, 100% AMD water; Treatment 3 = 1:100, 1 g QL and 100% AMD water; Treatment 4 (T4) = 2:100, 2 g QL and 100% AMD water; and Treatment 5 (T5) = 2:75:100, 2 g QL and 75% FA and 100% AMD water), a total of 40 and 36 metabolites were identified from Marykies and Royal cultivars, respectively. These include amino acids, organic acids, and aromatic amines (Table 1) below. Overall, the two cultivars shared similarity in the detected metabolites. However, adenosine monophosphate, cytidine, xanthine, lactic acid, and isocitric acid were only detected in the Marykies potato cultivar.

### 2.2. Effect of the Quicklime Treatment of AMD on the Two Cultivar Metabolomes Using LC-MS/MS

**Figure 1:**
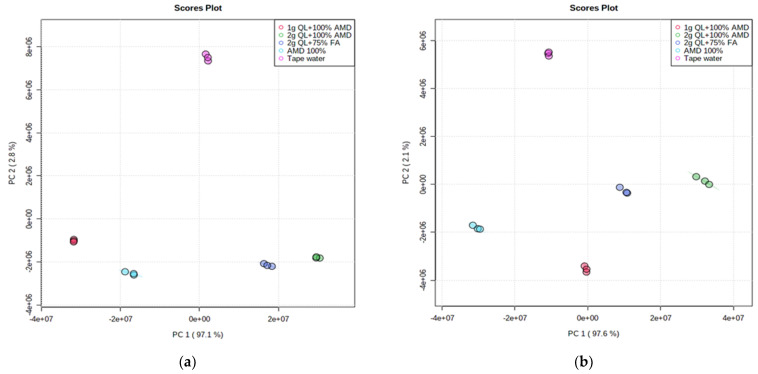
2D Principal component (PCA) analysis score plot from liquid chromatography linked to mass spectrometry data of samples from Marykies (**a**) and Royal (**b**) potato tuber samples. Different colors denoted different treatments: tap water (T1): purple, AMD 100% (T2): light blue, 1 g QL + 100% (T3): red, 2 g QL + 100% AMD (T4): green and 2 g QL + 75% FA (T5): dark blue, respectively.

The unsupervised Principal Component Analysis (PCA) approach for pattern recognition analysis was applied to the LC-MS chromatograms of Marykies and Royal potato tubers to give a comparative interpretation and visualization of metabolic differences between them amongst the five irrigation treatments used in this study. The PCA revealed that the control sample was clearly separated from the samples with AMD (both treated and untreated) along the PC2 axis, accounting for 2.8% and 2.1% of the Marykies and Royal cultivars, respectively (Figure 1a,b). Furthermore, along the PC1 axis, accounting for 97.1% and 97.6% of the Marykies and Royal cultivars, respectively, the 2 g QL + 100% AMD (T4: Green) and 2 g QL + 75% FA (T5: Dark blue) were well separated, indicating the impact of the treatments on the metabolite profiling in the two cultivars (Figure 1a,b). The resulting Scree plot explained by the PCs showed variance across the treatments used in this study (See Supplementary information online Figure S1). Treatments 2 and 3 were the same in T1, T4 and T5.

**Figure 2:**
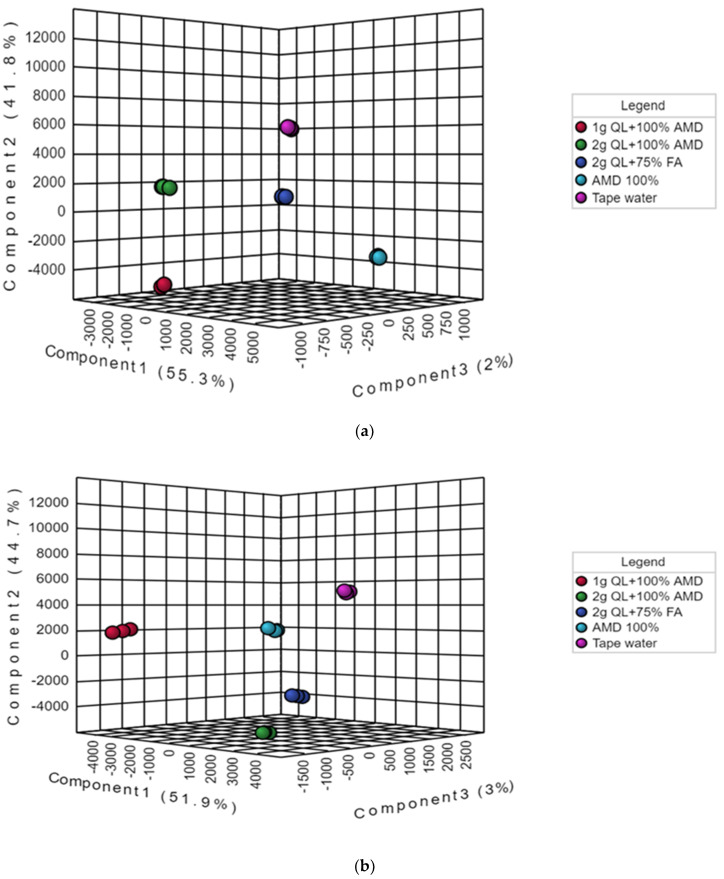
Partial least squares-discriminate analysis (PLS-DA) score plots of metabolic profiles from liquid chromatography linked to mass spectrometry data of samples from Marykies (**a**) and Royal (**b**) potato tuber samples. Different colors denote different treatments: tap water (T1): purple, AMD 100% (T2): light blue, 1 g QL + 100% (T3): red, 2 g QL + 100% AMD (T4): green and 2 g QL + 75% FA (T5): dark blue, respectively.

However, to obtain a higher level of treatment separation and a better understanding of the variables responsible for classification, a supervised PLS-DA was applied. The PLS-DA, in contrast to PCA, is a supervised approach that can categorize observations into groups based on the greatest predicted indicator variable [49,50]. Barker, in 2012 [51], employed statistical theory to demonstrate that PLS-DA was capable of accurate classification. The results showed a significant discrimination of the T1 (tap water: control) and the other treatments (T2–5) (Figure 2a,b). The treatments differentiated from each other on the first two components of the PLS-DA score plot by the principal component t(1) (51.9%), principal component t(2) (44.7%), and principal component t(3) (3%) for the Marykies cultivar, and principal component t(1) (55.3%), principal component t(2) (41.8%), and principal component t(3) (2%) for the Royal cultivar (Figure 2a,b). For the Marykies cultivar, the PLS-DA revealed five distinct groups, tapwater (T1) grouped at the top towards the right side of the PLS-DA score plot, while the 100% AMD (T2) and 1 g QL + 100% AMD (T3) aligned together in the middle towards the right and left, respectively, of the PLS-DA score plot. The 2 g QL + 100% AMD (T4) and 2 g QL + 75% FA (T5) aligned at the bottom towards the right of the PLS-DA score plot. A different alignment was observed for the Royal cultivar, with tapwater (T1) appearing at similar position as the Marykies cultivar in the PLS-DA score plot. The 2 g QL + 100% AMD (T4) and 2 g QL + 75% FA (T5) aligned in the middle towards the left and right, respectively, of the PLS-DA score plot. The 100% AMD (T2) and 1 g QL + 100% AMD (T3) aligned together at the bottom towards the right and left, respectively of the PLS-DA score plot. In addition, similar results to the PLS-DA score plot were observed in the PLS-DA S-plot for the Marykies cultivar, which also revealed 5 distinct groups (See Supplementary information online Figure S2). However, 100% AMD (T2) and 1 g QL + 100% AMD (T3) were aligned together at the bottom. A similar position was observed for 2 g QL + 100% AMD (T4) and 2 g QL + 75% FA (T5). For the Royal cultivar, the S-plot for 100% AMD (T2) and 1 g QL + 100% AMD (T3) aligned together at the bottom and 2 g QL + 100% AMD (T4) and 2 g QL + 75% FA (T5) aligned at the top, respectively. The PLS-DA model results for the Marykies cultivar showed a good fit test as designated by R^2^X = 0.55, R^2^Y = 0.99 and Q^2^X = 0.32 Q^2^Y = 0.99. While for the Royal cultivar, the model results were R^2^X = 0.67, R^2^Y = 0.99 and Q^2^X = 0.50 Q^2^Y = 0.99 (See Supplementary information online Table S1). The differences in the alignment of the different metabolites under the various treatments could be attributed to the variation in the physiological response of the cultivars under environmental stress [52,53]. Through metabolic alterations, plants can adjust their physiology to diverse situations [54]. According to [55], plants use a variety of metabolic adaption mechanisms to protect themselves from the harmful impacts of stress, and these systems can play an important role in plant adaptive mechanisms.

**Figure 3:**
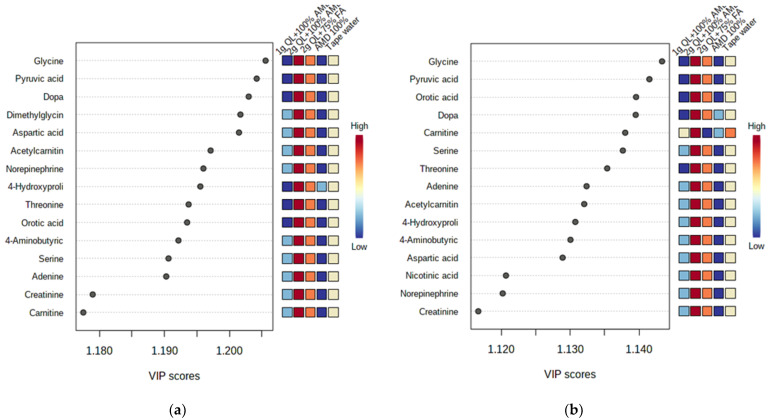
Discriminant metabolites identified by PLS-DA ranked by variable importance in projection (VIP) scores in component 1. The relative abundance of each metabolite from Marykies (**a**) and Royal (**b**) cultivars are indicated with a color code scaled from blue (low) to red (high). Tap water (T1); AMD 100% (T2); 1 g QL + 100% (T3); 2 g QL + 100% AMD (T4) and 2 g QL + 75% FA (T5), respectively.

The most important discriminant metabolites (identified by PLS-DA) ranked by variable importance in projection (VIP) scores in component 1 that delineated the metabolite abundance in the two cultivars impacted by the AMD and the treatments (Figure 3). For both cultivars, the 100% AMD (T2) and 1 g QL + 100% AMD (T3) treatments resulted in the minimal production of the identified metabolites (glycine, dopa, pyruvic acid, dimethylglycine, aspartic acid, acetylcarnitin, norepinephrine, 4-hydroxyproline, threonine, orotic acid, serine, adenine, creatinine, cartinine, and 4-aminobutyric acid) in comparison to the tapwater (T1) (Figure 3). The VIP scores that delineated the metabolites in component 1 were higher in the 2 g QL + 100% AMD (T4) and 2 g QL + 75% FA (T5) treatments for both cultivars when compared to the control (T1). This could imply that two treatments not only decontaminate the AMD but have the ability to initiate the production of the vital metabolites necessary for the growth of the potato, as was mentioned by [56] in the study of the growth and accumulation of heavy metals in wheat (*Triticum aestivum*), mung beans (*Vigna radiata*), and urad beans (*Vigna mungo*). The greater the impact of the metabolite as a distinguishing trait among cultivars, the higher the VIP score [57]. Only the metabolites having a VIP score of greater than one eere considered.

### 2.3. Hierarchical Cluster Analysis of Metabolomes in Two Cultivars of Potato under Irrigation with Treated AMD Water

**Figure 4:**
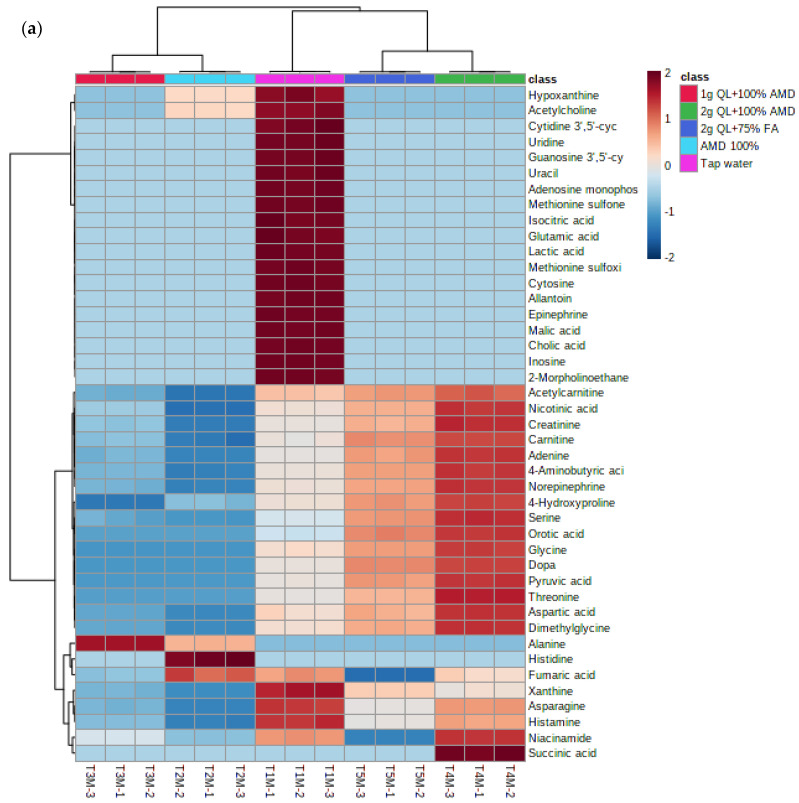

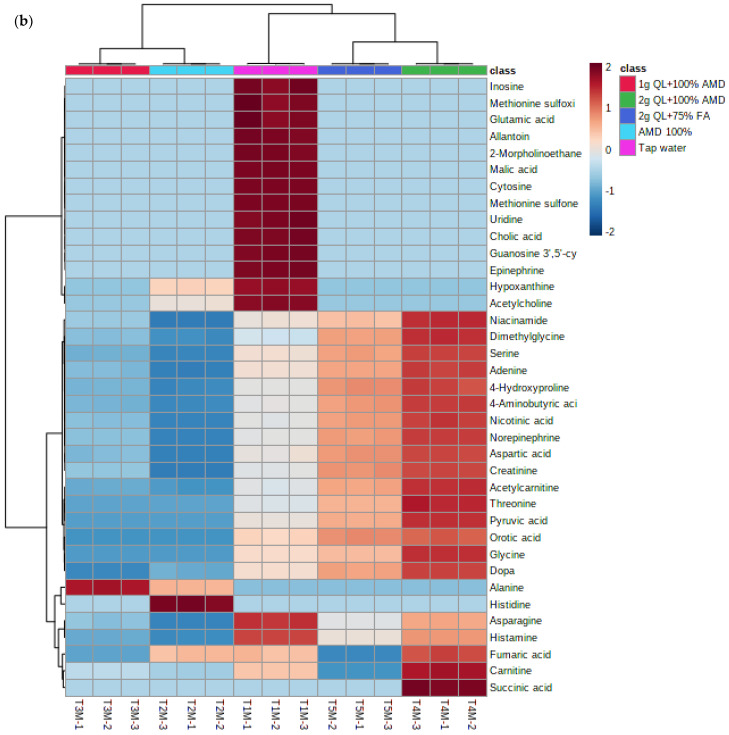
Heatmap of metabolites from liquid chromatography linked to mass spectrometry data of samples from Marykies (**a**) and Royal (**b**) potato tuber samples. Different colors denote different treatments: tap water (T1): purple, AMD 100% (T2): light blue, 1 g QL + 100% (T3): red, 2 g QL + 100% AMD (T4): green and 2 g QL + 75% FA (T5): dark blue, respectively.

The separation of metabolites is further revealed in the heatmap (Figure 4a,b) below. The heatmap revealed different groups of primary metabolites of two potato cultivars irrigated with treated AMD, indicating that potato metabolome patterns were dependent on treatment levels. The heatmap comprises of five clusters as per the treatments used in this study. The treatments 2 g QL + 100% AMD (T4) and 2 g QL + 75% FA (T5) clustered together, indicating the effect of the two treatments in the spatial distribution of synthesized metabolites for both cultivars (Figure 4). Further, AMD 100% (T2) and 1 g QL + 100% (T3) showed clustering, indicating similarities in the metabolites produced by the two cultivars, as the 1 g QL + 100% AMD (T3) may not have had strong impact in the treatment of the AMD, leading the plants to respond similarly in the production of metabolites under the two treatments. Similar results were confirmed by the clustering pattern shown as the dendrogram of both Marykies and Royal potato cultivars (See Figure S3).

## 3. Discussion

The two potato cultivars exhibited spatially distinct metabolomes under AMD conditions as well as the quicklime treated microenvironment. Metabolites that ranged from amino acids, organic acid, and aromatic amines showed differential spatial exudation and accumulation at the tuber level of the two potato cultivars. These results were consonant with those of several studies that reported the accumulation of higher quantity of amino acids, organic acids, sugars, and sugar alcohol are vital protective responses of plants in response to abiotic stress [58,59,60,61].

The partial least squares-discriminate analysis (PLS-DA) score plots, a supervised discriminant analysis, clearly delineated the metabolite profiles of the different treatments for both cultivars of potato. The discrimination of the metabolites accumulated by the tubers of the two cultivars of potato under the 100% AMD: (T2) condition indicated the spatial exudation of metabolites by the cultivars, possibly at the rhizospheric level, to promote the adaptability of the crops to the acidic conditions of the AMD as well as to the heavy metal toxicity. In agreement with our findings, Tan et al. 2021 [62] reported the exudation of diverse metabolites, including amino acids, linoleic acid, arginine, valine, leucine, and isoleucine at the rhizospheric level and tissue of *Brassica juncea* under Cd stress conditions. Under conditions of metal toxicity, plants coordinate the metabolic activities involved in plant growth and development, inducing higher levels of amino acids, their derivatives, and the organic acids necessary for their adaptation [63]. The close grouping of the 2 g QL + 100% AMD: (T4) and 2 g QL + 75% FA: (T5) treatment PLS-DA score plots provide an indication of the effects of the two AMD treatments on the metabolite exudation of the two cultivars of potato. This could be attributed to the effect of the treatments in the reduction of the contaminants present in the AMD, such as heavy metals, as well as to the stimulation effect of the quicklime/fly ash on the potato. Shanmugaraj et al. 2013 [64] reported the association of metabolites, such as organic acids, amino acids, peptides, glutathione, and phytochelatins, in the detoxification of heavy metal toxicity. Acid mine drainage water contains high concentrations of heavy metals. Therefore, during heavy metal stress, amino acids play important roles in metal binding, antioxidant defense, and signaling in plants [65,66,67]. Organic acids are other compounds that have been implicated in the defense against heavy metal stress [68]. This could account for the close grouping of the treated AMD water in the PLS-DA used as a source of irrigation of the potato, as any trace of heavy metals could trigger the exudation of similar metabolites. Furthermore, it is possible that the presence of the quicklime and the fly ash used in the treatment of the AMD might have induced the production of similar metabolites. However, further study is recommended to elucidate the direct impact of quicklime and fly ash on the metabolite profiles of the two potato cultivars.

In this study, the discrimination of metabolites using variable importance in projection (VIP) scores in component 1 analysis indicated a significant elevation in the abundance of glycine, dopa, pyruvic acid, dimethylglycine, aspartic acid, acetylcarnitin, norepinephrine, 4-hydroxyproline, threonine, orotic acid, serine, adenine, creatinine, cartinine, and 4-aminobutyric acid at the tuber level of potatoes under the 2 g QL + 100% AMD: (T4) and 2 g QL + 75% FA: (T5) treatments for both cultivars when compared to the tapwater control:(T1) and 100% AMD: (T2). These results imply that different metabolites can be stored in different tissues and cells depending on the function of the metabolites and the environmental stress that promoted their secretion [69]. As already alluded [55] to protect themselves from the damaging effects of stress, plants have a variety of metabolic adaption mechanisms, and these systems can play an important role in plant adaptive processes. Amino acids in plants contribute to the detoxification process by regulating ion transport, chelating ions, and nitrogen (N) metabolism under heavy metal stress [63]. The elevation in the relative abundance of the amino acids and other metabolites in the tubers of potato cultivars irrigated with the quicklime- and fly ash-treated AMD could be attributed to the response of the crops to heavy metal stress that could be present in the treated AMD water as well as to the impacts of the quicklime or fly ash, individually or synergistically, on the crops.

## 4. Materials and Methods

### 4.1. Study Area

The study was conducted at the UNISA Science campus, University of South Africa (Unisa), Johannesburg, Gauteng Province (S 26°10′30″ S, 27°55′22.8″ E). Acid mine drainage water samples were collected from Gold Mine in Gauteng, using sterile 50 L plastic containers (that had been cleaned with 20% sodium hypochlorite and UV-sterilized for one hour).

### 4.2. Experimental Design, Irrigation Treatments, Planting and Harvesting of Potato Cultivars

Prior to potato planting, water samples were accurately measured into 2 liter (L) containers. A total of five experimental treatments with different solution ratios (amount (g) of QL: percentage of fly ash (FA): percentage of AMD were used, as shown below. A total of five experimental treatments with different solution ratios (amount (g) of QL and FA percentage) were used, as shown below. Treatment 1(T1) = 0:0, tap water; Treatment 2(T2) = 0:100, 100% AMD water; Treatment 3 = 1:100, 1 g QL and 100% AMD water; Treatment 4 (T4) = 2:100, 2 g QL and 100% AMD water and Treatment 5 (T5) = 2:75:100, 2 g QL and 75% FA and 100% AMD water.

The analyzed quicklime-treated AMD waters (T1–T5) were used for irrigation for two potato cultivars, Royal and Marykies (Cultivars) planted in the greenhouse. The factorial experiment involved randomized blocks which comprised six pots (2 × 5), in which potato tubers of almost equal diameters between 30–60 mm were planted in each 20 × 20 cm pot. A mixture of 3:1:1 Culterra topsoil, vermiculite and river sand was used as a substrate. After planting, all the pots were immediately irrigated with tap water for 1 week until sprouts developed before application of the treatments. As previously stated, the irrigation treatments consisted of acid mine drainage that was improved with different quantities of quicklime. From emergence until crop maturity, irrigation with the various AMD treatments was applied every two days (senescence). An Irrometer Soil Moisture Meter (SN: 946,776) (Model 30–KTCD–NL) was used to accurately schedule irrigation. When the Irrometer reading was between 60 and 100 centibars, 500 mm irrigation water was applied to all experimental pots every cycle.

The experiment was conducted for a period of 90 days after planting (DAP). At the end of the experiment, each potato cultivar was harvested. All harvested tubers were rinsed with tap water, dried with paper towels, and weighed. Fresh tubers for each potato cultivar were freeze-dried using a freeze dryer (Free Zone Plus 2.5 L Cascade Benchtop Freeze Dry System Vacutec, Kansas City, MO, USA) and then ground to a powder using a benchtop grinder and stored in glass vials below −50 °C for metabolomic analysis. The samples of each cultivar were denoted as T1 to T5 represent the treated water and metabolite samples.

### 4.3. Metabolomic Analysis

#### 4.3.1. Metabolite Extraction

The untargeted metabolomics analysis extraction in this study was performed following the protocol from [70]. For extraction, a total of 0.5 g of freeze-dried ground potato tuber was weighed in a 2 mL Eppendorf tube, then 1.5 mL of MeOH (75% MEOH/25% water) was added and mixed with a vortex mixture. The mixture was sonicated for five (5) minutes using a BRANDSON 1800 (Darmstadt, Germany). The sonicated supernatant concentrate was then filtered through 0.2-micron syringe filters (Sartorius Minisart RC 4) with a 1 mL pipette. The supernatant-filtered concentrate was then centrifuged in an Eppendorf tube (Centrifuge 5424, Modderfontein, South Africa) at 10,000 revolutions per minute (rpm). The supernatant concentrates of the 72 samples were then transferred in HLPC vials to the LCMS-8040 triple quadrupole mass spectrometer (Shimadzu, Kyoto, Japan) for analysis. Seven hundred microliters of the supernatant concentration of 90 samples (45 for each cultivar across the treatments) were then pipetted into HPLC vials, which were then sealed with metals caps with rubber septas, secured with a crimper.

#### 4.3.2. Metabolite Detection

The separation analysis was carried out using a Dionex Ultimate 3000 UHPLC system (Dionex Softron GmbH, Germering, Germany) equipped with an electrospray ion source (ESI). The separation and detection of metabolites was achieved using a reversed-phase C18 analytical column of 100 mm × 2.1 mm and 1.7 µm particle size (Acquity UPLC^®^ BEH, Waters, Ireland), maintained at 35 °C. The injection volume was 2 µL. The mobile phase consisted of 0.1% formic acid in water (solvent A) and LC-MS grade methanol (solvent B), at a flow rate of 0.3 mL/min. The gradient elution applied was: 85% A: 15% B to 65% A: 35% B in 4 min, changed to 50% A: 50% B for 2 min, then to 20% A: 80% B for 1 min, and back to the initial ratio (85% A: 15% B to 65% A: 35% B) for 0.5 min. Furthermore, the UHPLC system was interfaced with a Xevo G2 QT of water, then applied using the source-ESI positive and negative modes; capillary voltage-3 kV; cone voltage 30 V; calibration-sodium formate; lock spray-leucine enkephalin. The data acquisition was performed using LabSolutions software LC-MS Ver.5.82 (Shimadzu, Kyoto, Japan) and LabSolutions database, at an acquisition rate and range of 30 spectra/s and *m*/*z* 60–1500, respectively. To compare the treatments, the quantities of metabolites were plotted in STATISTICA (StaSoft Inc., Tulsa, OK, USA, 2011) package. The extracts were analyzed by reverse phase LC-MS for their metabolomic contents. The MS analysis was carried out in the electron spray (ESI) positive and negative modes used for checking mass accuracy. A method followed by [71] was adopted, whereby peak intensity-showing LCMS-8040 triple quadrupole mass spectrometer intensities represented the quantities of the metabolites, varying with the treatments. The denotations T1 to T5 represent metabolites in the various treated tuber samples.

#### 4.3.3. Data Processing and Statistical Analysis

For this study, data processing and statistical analysis were carried out by MetaboAnalyst 5.0 software (https://www.metaboanalyst.ca accessed on 10 February 2022) [72] an online statistical package. For statistical analyses, peak areas were taken into consideration. Before using various chemometrics methods, the data collected must be pre-processed. For this study, this processing included data alignment [73], normalization [74] scaling (pareto) and transformation (logarithm transformation) [75,76]. The top metabolites for tapwater versus treated AMD water were depicted on heat maps based on the Pearson distance measured, using the Ward clustering technique to visualize relative levels. Multivariate tests such as partial least-squares discriminant analysis (PLS-DA) and principal component analysis (PCA) were used to display significant metabolites among the studied groups. The PLS-DA method is a supervised method for analyzing huge data sets. With a significance level of *p* ≤ 0.05, the variable importance in projection (VIP) scores were used to rank the overall impact of each variable. The links between metabolites were discovered using dendrogram analysis. PLS-DA, VIP scores, and heat maps were used to identify the key metabolites.

## 5. Conclusions

This study elucidated the impact of AMD and quicklime/fly ash-treated AMD on the spatial exudation and accumulation of metabolites at the tuber level of two potato cultivars. Overall, the results showed that the AMD and the treatments influenced the exudation and accumulation of metabolites in the tubers of the two cultivars, with subtle differences in exudation within the two cultivars. The elevation in the abundance of glycine, dopa, pyruvic acid, dimethylglycine, aspartic acid, acetylcarnitin, norepinephrine, 4-hydroxyproline, threonine, orotic acid, serine, adenine, creatinine, cartinine, and 4-aminobutyric acid in the tubers of crops irrigated with treated AMD water imply their role in the maintenance of the health and growth of the two crops, as these metabolites are believed to be the protective responses of crops to environmental stress. Despite the role of quicklime and fly ash in the removal of the heavy metal constituent of AMD water, their presence in the irrigated water was observed to trigger the exudation of metabolites in the crops, as the crops might have recognized the chemicals as stress condition. This was evident in the lower abundance of glycine, dopa, pyruvic acid, dimethylglycine, aspartic acid, acetylcarnitin, norepinephrine, 4-hydroxyproline, threonine, orotic acid, serine, adenine, creatinine, cartinine, and 4-aminobutyric acid in the 100% AMD (T2) crops, as compared to the controls (T1). However, further studies are recommended to evaluate the direct impact of quicklime and fly ash on metabolite exudation in potato cultivars.

## Figures and Tables

**Table 1:** Metabolites identified from a methanol tissue extract of potato tubers.

Irrigation Treatments Used	Metabolites		
	Amino Acids	Organic Acids	Aromatic Amines
Marykies			
Treatment 1 (T1)	Acetylcarnitine, Acetylcholine, Adenosine monophosphate, Adenine, Allantoin, Asparagine, Aspartic acid, Carnitine, Creatinine, Cytidine, Cytosine, Dimethylglycine, Epinephrine, Glutamic acid, Glycine, Guanosine 3′,5′-cyclic monophosphate, Histamine, Hypoxanthine, Inosine, Methionine sulfone, Methionine sulfide, Niacinamide, Norepinephrine, Serine, Threonine, Uridine, Xanthine, 4-Aminobutyric acid, 4-Hydroxyproline	Cholic acid, Fumaric acid, Isocitric acid, Lactic acid, Malic acid, Nicotinic acid, Orotic acid, Pyruvic acid, and 2-Morpholinoethanesulfonic acid	Dopa
Treatment 2 (T2)	Acetylcarnitine, Acetylcholine, Adenine, Alanine, Asparagine, Aspartic acid, Carnitine, Creatinine, Histamine, Histidine, Hypoxanthine, Niacinamide, Norepinephrine, Serine, 4-Aminobutyric acid, 4-Hydroxyproline	Fumaric acid, Nicotinic acid	
Treatment 3 (T3)	Acetylcarnitine, Adenine, Alanine, Asparagine, Aspartic acid, Carnitine, Creatinine, Dimethylglycine, Dimethylglycine, Histamine, Niacinamide, Norepinephrine, Serine, Xanthine, 4-Aminobutyric acid, 4-Hydroxyproline	Fumaric acid, Nicotinic acid	
Treatment 4 (T4)	Acetylcarnitine, Adenine, Asparagine, Aspartic acid, Carnitine, Creatinine, Dimethylglycine, Glycine, Histamine, Niacinamide, Norepinephrine, Serine, Threonine, 4-Aminobutyric acid, 4-Hydroxyproline	Fumaric acid, Isocitric acid, Nicotinic acid, Orotic acid, Pyruvic acid	Dopa
Treatment 5 (T5)	Acetylcarnitine, Adenine, Asparagine, Aspartic acid, Carnitine, Creatinine, Dimethylglycine, Glycine, Histamine, Norepinephrine, Serine, Threonine, Xanthine, 4-Aminobutyric acid, 4-Hydroxyproline	Isocitric acid, Nicotinic acid, Orotic acid, Pyruvic acid	Dopa
Royal			
Treatment 1 (T1)	Acetylcarnitine, Ace-tylcholine, Adenine, Allantoin, Asparagine, Aspartic acid, Carnitine, Creatinine, Cytosine, Dimethylglycine, Epinephrine, Glutamic acid, Glycine, Guanosine 3′,5′-cyclic monophosphate, Histamine, Hypoxanthine, Inosine, Methionine sulfone, Me-thionine sulfide, Niacinamide, Norepinephrine, Serine, Threonine, Uridine, 4-Aminobutyric acid, 4-Hydroxyproline	Cholic acid, Fumaric acid, Malic acid, Nicotinic acid, Orotic acid, Pyruvic acid, Succinic acid and 2-Morpholinoethanesulfonic acid	Dopa
Treatment 2 (T2)	Acetylcarnitine, Acetylcholine, Adenine, Asparagine, Aspartic acid, Carnitine, Creatinine, Dimethylglycine, Histamine, Histidine, Hypoxanthine, Niacinamide, Norepinephrine, Serine, 4-Aminobutyric acid, 4-Hydroxyproline	Fumaric acid, Nicotinic acid	
Treatment 3 (T3)	Acetylcarnitine, Adenine, Alanine, Asparagine, Aspartic acid, Carnitine, Creatinine, Dimethylglycine, Histamine, Niacinamide, Norepinephrine, Serine, 4-Aminobutyric acid, 4-Hydroxyproline	Fumaric acid, Nicotinic acid	
Treatment 4 (T4)	Acetylcarnitine, Adenine, Asparagine, Aspartic acid, Carnitine, Creatinine, Dimethylglycine, Glycine, Histamine, Niacinamide, Norepinephrine, Serine, Threonine, 4-Aminobutyric acid, 4-Hydroxyproline	Fumaric acid, Nicotinic acid, Pyruvic acid, Succinic acid	Dopa
Treatment 5 (T5)	Acetylcarnitine, Adenine, Carnitine, Creatinine, Dimethylglycine, Glycine, Histamine, Niacinamide, Norepinephrine, Serine, Threonine, 4-Aminobutyric acid, 4-Hydroxyproline	Nicotinic acid, Pyruvic acid	Dopa

TM (1–5) TM1 represent metabolites on treated tuber samples across (un)treated AMD water used for irrigation.

## Data Availability

Not applicable.

## Supplementary Materials

The following data are available online https://www.mdpi.com/article/10.3390/metabo12030221/s1. Figure S1: Scree plots for both Marykies and Royal cultivars, showing the variance across the treatments that were explained by PCs. Figure S2: PLS-DA s-plot for both Marykies and Royal cultivars. Figure S3: Clustering pattern shown as the dendrogram of potato cultivars Marykies and Royal. Different colors denote different treatments: tap water (T1): purple, AMD 100% (T2): light blue, 1 g QL + 100% (T3): red, 2 g QL + 100% AMD (T4): green and 2 g QL + 75% FA (T5): dark blue, respectively. Table S1: PLS-DA models for Marykies and Royal potato cultivars.
