# Supplementary material for: LC-MS Based Metabolomics Analysis of Potato (Solanum tuberosum L.) Cultivars Irrigated with Quicklime Treated Acid Mine Drainage Water
Source: Metabolites. 2022 Mar 2;12(3):221. doi: 10.3390/metabo12030221 (PMC8952287; doi:10.3390/metabo12030221)
Supplement: Supplementary file 1 [file metabolites-12-00221-s001.zip › metabolites-1552218-supplementary.pdf]

Supplementary Information for LC-MS Based Metabolomics Analysis of Potato (Solanum tuberosum L.) Cultivars Irrigated with Quicklime Treated Acid Mine Drainage Water study.

Marykies data Scree plot

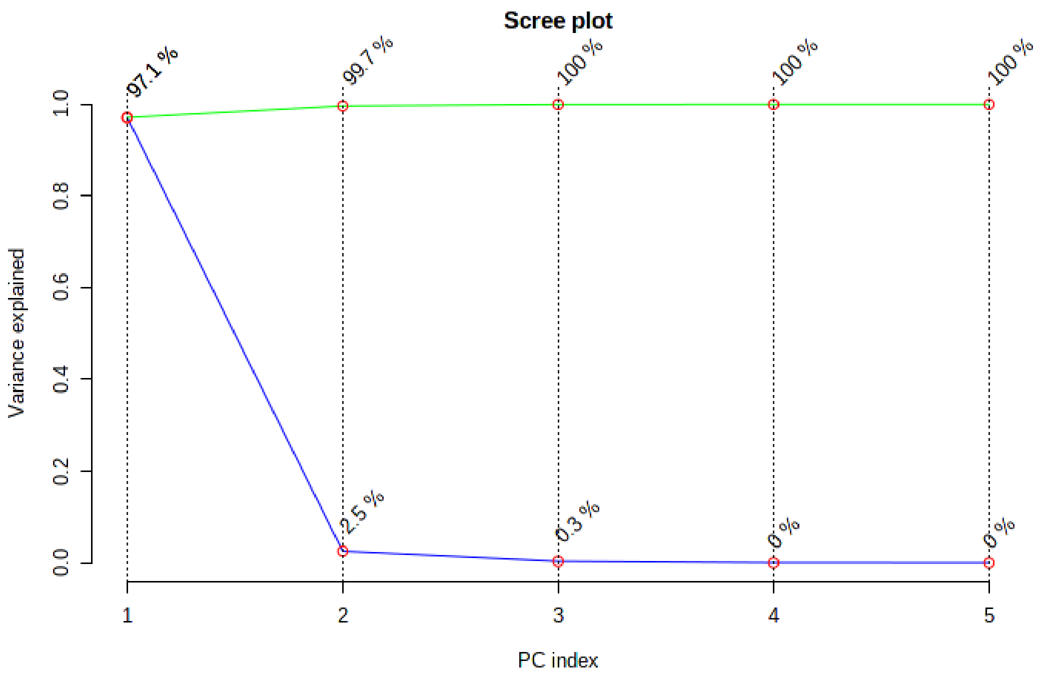

Royal Scree plot

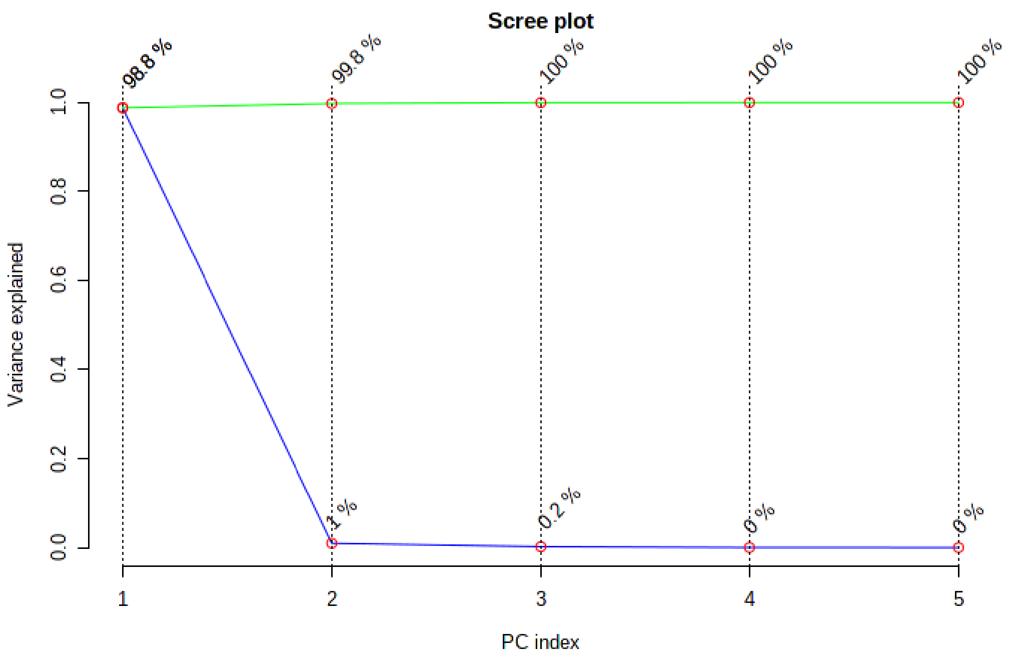

Figure S1: Scree plot showed that the variance across the treatments that were explained by PCs. It presented that Treatment 2 and 3 were the same as compared to T1, T4 and T5.

Marykies S plot for PLSDA

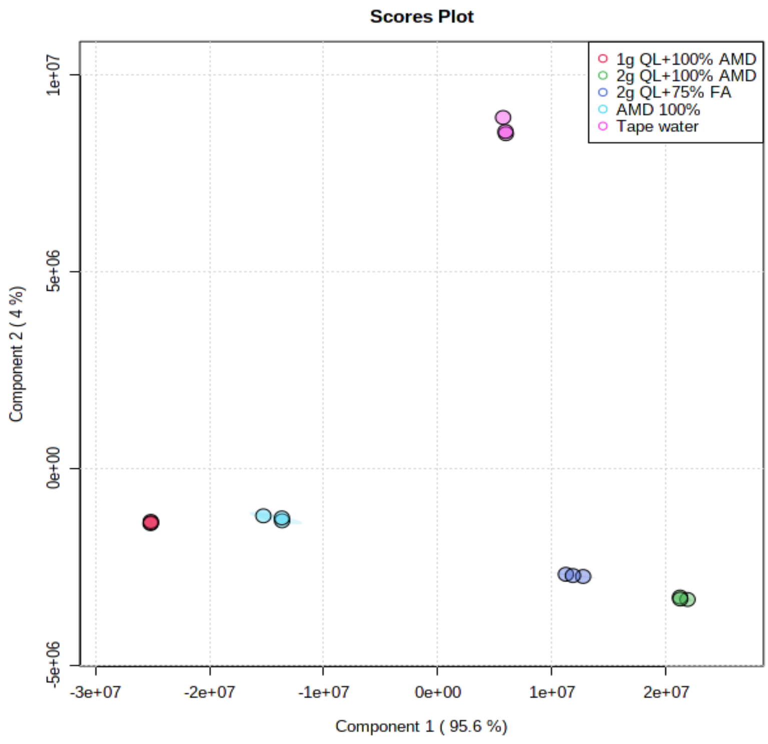

Royal S plot for PLSDA

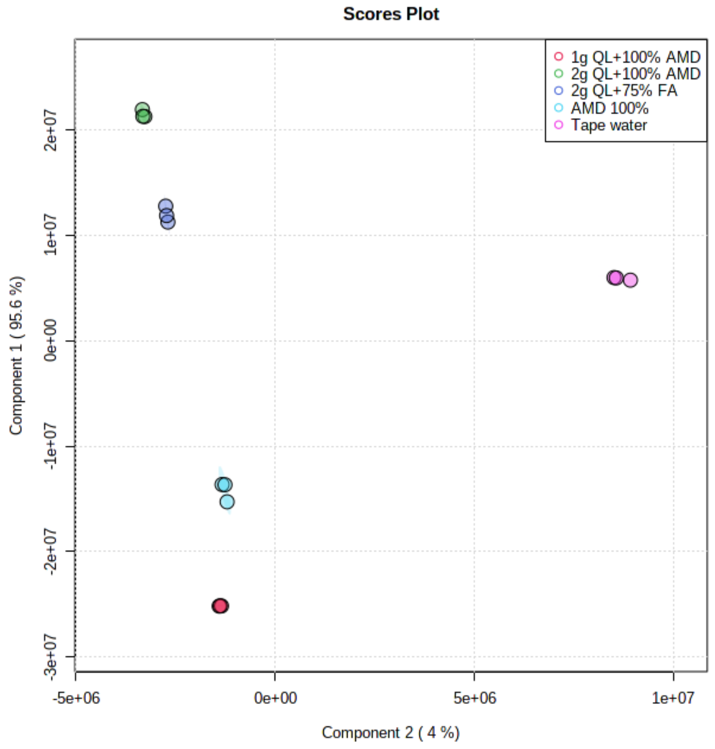

Figure S2: PLS-DA S-plot for Marykies and Royal cultivars. Tap water (T1): Purple, AMD 100% (T2): Light blue, 1g QL + 100% (T3): Red, 2g QL + 100% AMD (T4): Green and 2g QL + 75 % FA (T5): Dark blue respectively.

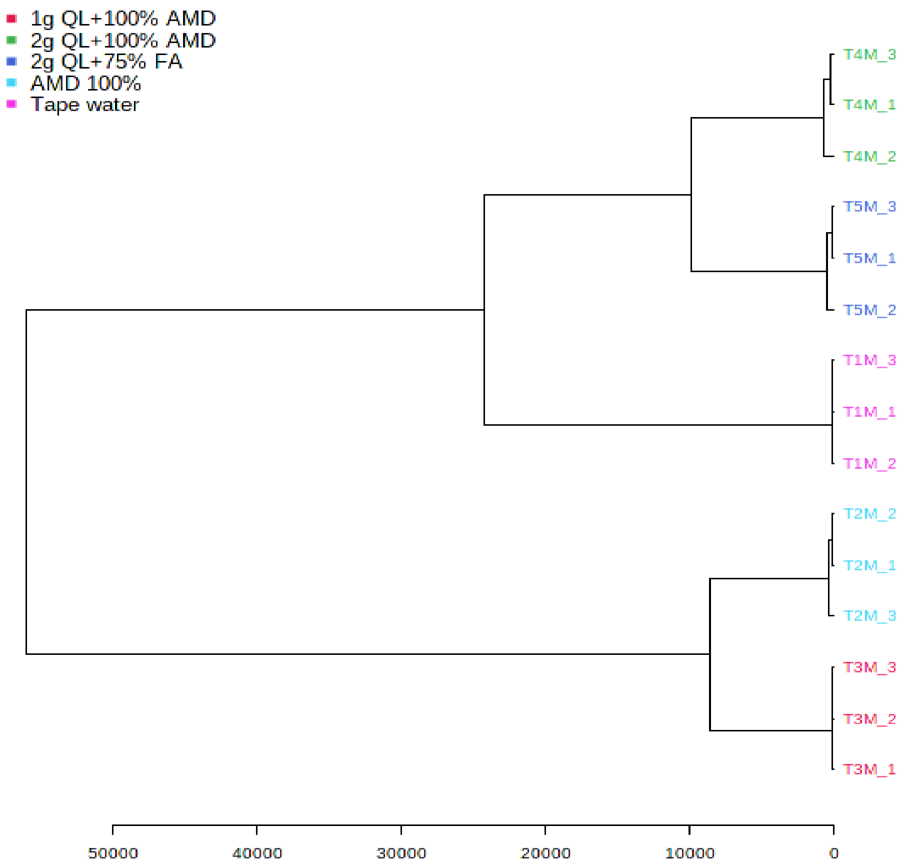

Figure S3: Clustering pattern shown as the dendrogram of potato cultivars Marykies and Royal. Different colours denoted different treatments: Tap water (T1): Purple, AMD 100% (T2): Light blue, 1g QL + 100% (T3): Red, 2g QL + 100% AMD (T4): Green and 2g QL + 75 % FA (T5): Dark blue respectively.

Table S1. PLS-DA model results for both Marykies and Royal cultivars.

Marykies model

**Measure**	**1 Comps**	**2 Comps**	**3 Comps**
Accuracy	0.0	0.2	0.8
R^2	0.047349	0.55227	0.99757
Q^2	-0.6121	0.32094	0.99577

Royal model

**Measure**	**1 Comps**	**2 Comps**	**3 Comps**
Accuracy	0.0	0.2	0.66667
R^2	0.031888	0.696	0.99894
Q^2	-0.17484	0.50579	0.99795
